# Functional Relations Modulate the Responsiveness to Affordances Despite the Impact of Conflicting Stimulus–Response Mappings

**DOI:** 10.3389/fpsyg.2017.01951

**Published:** 2017-11-07

**Authors:** Roberta Vastano, Martin Finn, Dermot Barnes-Holmes

**Affiliations:** Department of Experimental Clinical and Health Psychology, Ghent University, Ghent, Belgium

**Keywords:** affordance, IRAP, stimulus–response mappings, functional relations, conflict

## Abstract

The study investigated how conflicting stimulus–response mappings influenced affordance processing given a manipulation of the functional relations. Participants performed a task involving consistent–inconsistent stimulus–response mappings: *Implicit Relational Assessment Procedure* (IRAP). They were instructed to confirm or to deny a relation between words and tool-objects (consistent blocks) or to provide non-conventional responses (inconsistent blocks). The relations between stimuli could functionally match (e.g., Kitchen – Spatula) or not (e.g., Kitchen – Hammer), as well as the spatial relations (e.g., a match or a mismatch between participants’ hand response and the tool-object orientation). The results showed faster reaction times (RTs) when functional relations between stimuli matched both in consistent and inconsistent blocks. Differences in RTs and accuracy between consistent and inconsistent blocks were only found when the functional relation between stimuli matched. No modulation of the performance was observed for mismatching functional relations and spatial relations between blocks. These results support the hypothesis that the responsiveness to affordances is strongly modulated by matching functional relations, despite the impact of conflicting stimulus–response mappings.

## Introduction

The concept of affordance was first proposed by Gibson (1979) to refer to the action possibilities *automatically* evoked by the environment. Later work extended the concept of affordance to incorporate so-called micro-affordances (Ellis and Tucker, 2000) by indicating that the observation of graspable objects (tools) evokes one or more potential motor acts and that the simple sight of this particular category of objects automatically engages sensorimotor areas (Buccino et al., 2009; Cardellicchio et al., 2011; Proverbio et al., 2011; Goslin et al., 2012).

At the behavioral level, the automatic activation of affordances has been investigated in simple categorization tasks, by using stimulus–response compatibility paradigms mostly related to the spatial relation between the objects and the participants’ responses. For instance, participants are asked to classify objects according their category (tool or kitchen utensil, natural or artificial). The results of these tasks show faster reaction times (RTs) when the participant’s hand response is congruent with the orientation of the object’s handle (Tipper et al., 2006; Goslin et al., 2012); or when the required responses (i.e., precision or a power grip) are congruent with the relevant responses for interacting with the object [i.e., to grasp a small object or a large object (Ellis and Tucker, 2000; Tucker and Ellis, 2001)]. Automatic activation of affordances also has been investigated by using language. For instance reading function verbs (e.g., “to pour”) induced faster RTs when participants decided if the object–verb combination was appropriate, compared to when observation verbs (e.g., “to look at”) were presented. This effect was only present when the objects were presented within peripersonal (reachable) space, compared to extrapersonal space (non-reachable) (Costantini et al., 2011).

Taken together, these studies indicate that affordances are context-dependent mechanisms. In the same way, when we interact with such objects we are also influenced by the functional context given by the presence of paired objects (e.g., spatula – *pan*) (Yoon et al., 2010; Borghi et al., 2012) or by the functional environment (e.g., spatula – *kitchen*) (Wokke et al., 2016). For example, imagine you want to hang a picture and that you need a hammer—typically, you may go to look in the garage rather than in the kitchen. Based on this line of reasoning, a recent study used an ecological approach to test the effect of the environment on affordances (Wokke et al., 2016). The authors placed the participants in a real kitchen or a real workshop and told them to answer as fast as possible, by pressing a key when the target (any kitchen object or any tool) appeared on the screen and to inhibit their answer when a red “X” appeared (stop-task). The results showed faster RTs on go-trials, exclusively for the objects corresponding to the environment in which participants were situated (i.e., the observation of kitchen utensils when participants were placed in the kitchen). This finding highlights the importance of the context on affordance activation when a stimulus matches with the functionality of the environment. Relatedly, faster RTs have been also found when participants have to decide if two objects are usually used together and so functionally related (i.e., a spatula and a pan) compared to trials when the objects are unrelated (i.e., a bottle and a scissor) (Borghi et al., 2012). Thus, the functional relation between the observed items and the context denotes a salient meaning that automatically activates related motor knowledge.

However, previous studies have tended to focus their attention only on the simple comparison between congruent and incongruent functional contexts on affordance effects (Yoon et al., 2010; Borghi et al., 2012; Wokke et al., 2016), or on stimulus–response compatibility only related to spatial relations (Tucker and Ellis, 1998, 2001; Ellis and Tucker, 2000; Phillips and Ward, 2002). Whether and how a conflict induced by an inconsistent stimulus–response mapping influences the responsiveness to affordance within the framework of functional relations, thus remains an open question. Therefore, we asked if affordances activation within the framework of functional relations is context-dependent and would thus be modulated by such conflict.

In an inconsistent stimulus–response mapping, a dominant response has to be inhibited in support of an unusual response (overlearned relationship). As a result of the conflict, the RTs are usually slower compared to a consistent situation where the conflict does not occur. An example of a task that involves consistent–inconsistent stimulus–response mappings is the *Implicit Relational Assessment Procedure* (IRAP) (Barnes-holmes et al., 2010) which operates by requiring competing patterns of responses across blocks of trials. The IRAP presents a series of screens where contrasting categories of label and target stimuli are randomly combined into pairs. In consistent blocks, participants are required to confirm relationships between label and target stimuli considered in a congruent match (e.g., “Garage” – “Hammer” - “True”) and deny relationships between label and target stimuli considered mismatching (e.g., “Kitchen” – “Pliers” - “False”). In inconsistent blocks, participants are required to respond in the opposite manner by confirming mismatching relations or denying matching relations (e.g., “Kitchen” – “Pliers” - “True”; “Garage” – “Hammer” - “False”). In the current study, we aimed to test the effect of consistent–inconsistent stimulus–response mappings, created by the IRAP, on affordance activation given a matching–mismatching functional context.

Participants observed images of kitchen utensils or tools (with left or right facing handles) at the center of the screen and simultaneously on the top of the screen a label (a word) appeared. The word could represent a context functionally congruent or incongruent with the image that appeared on screen; for instance the word “Kitchen” and the object “Spatula” (matching functional context) or the word “Kitchen” and the object “Hammer” (mismatching functional context). During consistent blocks of trials, participants were required to relate the observed items in accordance with verbal convention (e.g., “Kitchen” – “Spatula” - “True”). During inconsistent blocks, the non-conventional response was required (e.g., “Kitchen” – “Spatula” - “False”).

In accordance with previous experimental evidence (Borghi et al., 2012; Wokke et al., 2016), we hypothesized that the activation of the affordance for the observed objects will be sensitive to the context (in our case, given by the presence of the words). That is, the matching functional context (e.g., “Kitchen” – “Spatula”) should induce faster RTs compared to the mismatching functional context (e.g., “Kitchen” – “Hammer”). Given that participants were required to respond with their left and right hands, we also predicted faster RTs when the orientation of the handle of the object was compatible with the participants’ hand response.

Finally, we reasoned that if the inconsistent stimulus–response mapping induces a conflict, then it could interfere with the affordance effects by producing slower RTs, compared to the consistent stimulus–response mapping. This effect could also be larger for the mismatching functional context, given the presence of an additional conflict related to the incongruent context (i.e., “Kitchen” – “Hammer”). However, considering the automatic activation of the affordance effects (Tucker and Ellis, 1998; Ellis and Tucker, 2000; Proverbio et al., 2011; Goslin et al., 2012), it is possible that the stimulus–response mapping variable would have little or no impact on affordance, and thus the functional context would dominate any stimulus–response mapping effect.

## Materials and Methods

### Participants

Twenty-three right-handed participants (five males, mean age 25.5 years; *SD*: 7.1 years) completed the current study in return for €5 payment. All participants were recruited via Ghent University’s online recruitment system. The study was approved by the ethics committee of the Department of Experimental, Clinical, and Health. All participants gave written informed consent in accordance with the principles of the Helsinki Declaration.

### Stimuli and Procedure

Subjects were seated at a distance of 60 cm from the computer monitor (refresh rate 60 Hz, using a monitor of 38 cm × 22 cm with a resolution of 1920 × 1080). Experimental stimuli consisted of label and image stimuli. Label stimuli were the words “Kitchen” and “Garage,” presented on the top of the monitor. Target stimuli (six different pictures of 700 × 600 pixels) were pictures of items regularly found in a kitchen (e.g., a spatula) or in a garage (e.g., a hammer). The pictures were presented at the center of the monitor, the graspable part of the objects was tilted at a 45° angle in order to point toward the participants’ hands. Each picture was presented with left or right handle orientation (compatible or incompatible with the grasping hand). Simultaneously with the appearance of the label and target stimuli, the response options (“True” and False”) were presented at the bottom left and right of the monitor. Participants were instructed to observe the stimuli and to relate the stimuli together by choosing “True” or “False.”

Each trial started with a fixation cross for a variable interval of 1000–1500 ms, then the stimuli (the label and the image) appeared (with a duration until participants’ response) and on each trial participants selected one of the two response options by using their left (pressing the key “d” on the keyboard) or right hand (pressing the key “k” on the keyboard). An inaccurate response produced a red “X” beneath the target stimulus that was only removed after the accurate response had been selected; this procedure allowed participants to learn implicitly the correct relationship between the stimuli. Failure to select a response within 2000 ms of stimulus presentation resulted in a red exclamation point appearing beneath the target stimulus. After the participants’ response and an inter trial interval of 1000 ms, the next trial started.

**FIGURE 1:**
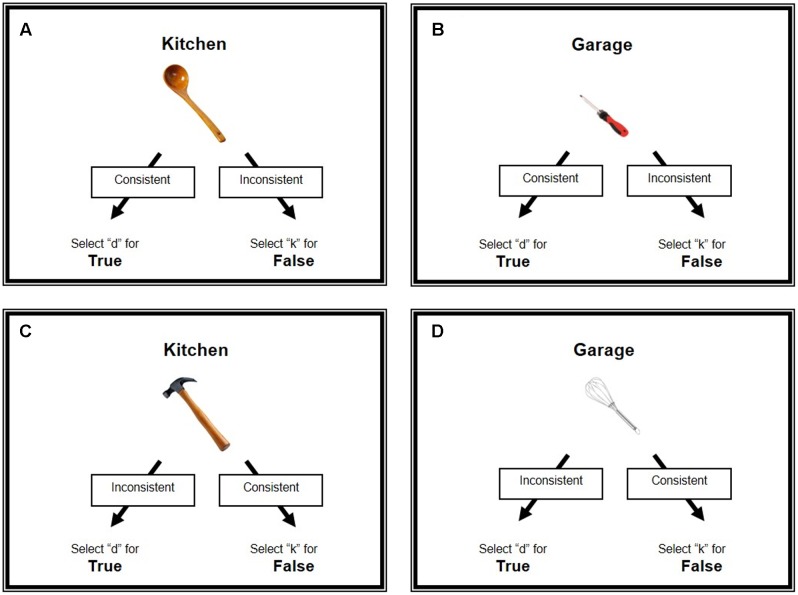
Experimental task. The figure shows an example of both functional contexts (Kitchen and Garage) for consistent and inconsistent stimulus–response mappings. Possible combinations: matching functional context **(A)** (Kitchen – Spoon); **(B)** (Garage – Screwdriver); mismatching functional context; **(C)** (Kitchen – Hammer); **(D)** (Garage – Whisk). Consistent and inconsistent labels indicate the trial type (e.g., consistent: conventional responses were required; inconsistent: non-conventional responses were required). Participants observed images of kitchen utensils and object-tools at the center of the screen, with their graspable part oriented toward left or right. In parallel with the images, a word appeared on the top of the screen. The word could be related to “Kitchen” or “Garage” environment. In consistent trials, participants confirmed or denied the relation between the observed items. For instance, “Kitchen” – “Spoon” - “True”; “Kitchen” – “Hammer” - “False.” In inconsistent trials, participants were required to provide an opposite response that is to answer in a non-conventional way. For instance, “Kitchen” – “Spoon” - “False”; “Kitchen” – “Hammer” - “True.” Participants provided their answers by pressing the letters “d” or “k.”

The current IRAP commenced with a consistent block of trials, and thereafter alternated between inconsistent and consistent blocks of trials. In consistent blocks, participants learnt to confirm conventional relationships between the label and target stimuli (“Garage” – “Hammer” - “True”) and deny non-conventional relationships (“Kitchen” – “Pliers” - “False”). During inconsistent blocks, participants were told that the task rules were switched and so they learnt to respond in the opposite manner (e.g., “Garage” – “Hammer” - “False”; “Kitchen” – “Pliers” - “True”) (**Figure 1**).

The design had three factors: Consistence (two levels: consistent vs. inconsistent blocks of trials), Functional Congruency (two levels: matching and mismatching functional context, provided by the conventional/non-conventional relation between labels and targets), and the Hand Response (compatible or incompatible with the handle orientation).

The experiment consisted of 384 randomized trials in total: with 192 consistent and 192 inconsistent trials; 96 trials for each matching and mismatching functional context that resulted in 48 for each orientation of the handle (compatible or incompatible), with 24 trials for each response option (“True” – “False”).

Each participant was presented with four pairs of blocks of trials. The first block in each pair presented combinations of label and target stimuli that required consistent responses; the second block in each pair required inconsistent responses. This resulted in a total of eight blocks (four consistent and four inconsistent) of 48 fully randomized trials. The position of the response options, “True” and “False,” alternated across each pair of blocks. For example, “True” appeared on the bottom left and “False” on the bottom right for the first pair of blocks and then for the second pair of blocks these positions switched (“True” on the right and “False” on the left). This variable was counterbalanced across participants.

Consistent with standard practice when using the IRAP, participants were provided with a practice phase, which required that they achieve a minimum of 80% accuracy and a maximum mean latency of 2000 ms on single pair consistent and inconsistent blocks. The task was implemented in E-prime 2.0 Professional software (Psychology Software Tools, Pittsburgh, PA, United States). The duration of the whole experiment was approximately 30 min.

### Data Analysis

We performed two separate 2 × 2 × 2 repeated measures analysis of variance (ANOVA) one for RTs and another for error rates (calculated as the percentage of incorrect responses). The three within-participant factors were: Consistence (two levels: consistent and inconsistent), Functional Congruency (two levels: matching and mismatching functional context), and Hand Response compatibility (hand compatible and hand incompatible with the orientation of the object handle). Significant effects found in the ANOVA were followed by Newman–Keuls corrected *post hoc* tests; alpha level was fixed at 0.05 for all statistical tests.

## Results

The ANOVA on RTs revealed a main effect for Consistence, *F*(1,22) = 12.89; *p* = 0.001; $\eta_{p}^{2}$ = 0.36, with faster RTs in consistent (*M* = 908 ms; *SD* = 150 ms) compared to the inconsistent condition (*M* = 954 ms; *SD* = 159 ms). There was also a significant main effect of Functional Congruency, *F*(1,22) = 74.65; *p* < 0.001; $\eta_{p}^{2}$ = 0.77. The matching functional context (*M* = 890 ms; *SD* = 143) led to faster RTs compared to the mismatching functional context (*M* = 972 ms; *SD* = 159 ms). The main effect of the hand response was not significant *F*(1,22) = 0.127; *p* = 0.724; $\eta_{p}^{2}$ = 0.005; (hand compatible: *M* = 930 ms; *SD* = 146 ms; hand incompatible: *M* = 933 ms; *SD* = 145 ms).

**FIGURE 2:**
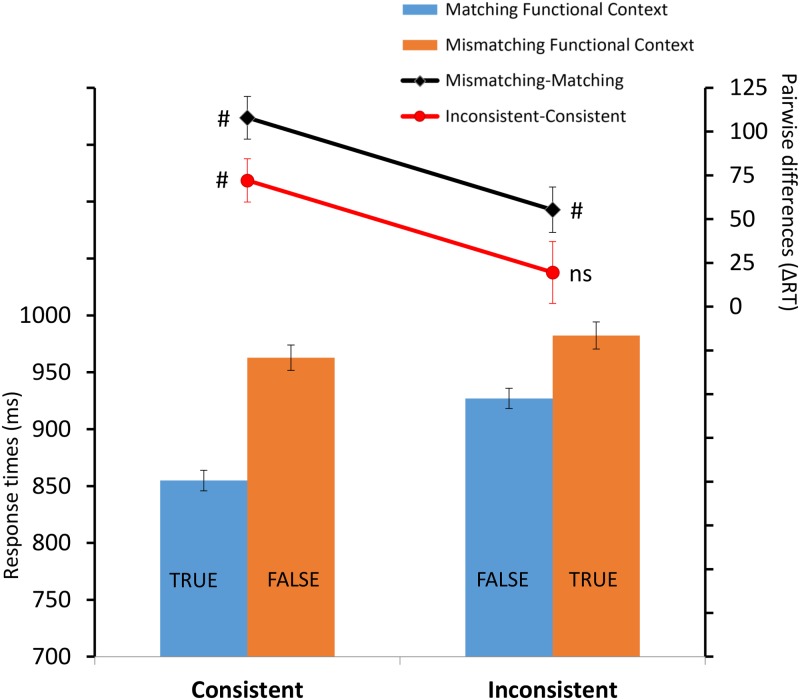
The bar plot shows the mean of response times as a function of X (consistent vs. inconsistent) and Y (matching functional context vs. mismatching functional context) factors. Error bars indicate within-subjects SEM (Morey, 2008). The black and red line plots show, respectively, the functional context effect (i.e., the mismatching functional context – matching functional context pairwise difference) in consistent and inconsistent conditions and the consistency effect (i.e., the inconsistent–consistent pairwise difference) in matching functional context and mismatching functional context conditions. The corresponding error bars indicate the SEM of the pairwise differences (Franz and Loftus, 2012). (#) indicates consistency and functional context effects that were significantly (*p* < 0.05) different from 0; (ns) indicates non-significant effects. TRUE and FALSE represent the two response options.

Most importantly, a significant interaction between Consistence and Functional Congruency was found *F*(1,22) = 9.99; *p* = 0.004; $\eta_{p}^{2}$ = 0.3 (**Figure 2**). *Post hoc* comparisons revealed that in the consistent condition (when we compared the response options “True” vs. “False”) the matching functional context induced faster RTs (*M* = 854 ms; *SD* = 131 ms) compared to the mismatching context (*M* = 962 ms; *SD* = 152 ms) (*p* < 0.001). The same pattern of RTs was also observed in the inconsistent condition (when we compared the response options “False” vs. “True”), with faster RTs in the matching functional context (*M* = 926 ms; *SD* = 149 ms) compared to mismatching (*M* = 982 ms; *SD* = 168 ms) (*p* < 0.001).

No significant effect was observed in the mismatching functional context between consistent (*M* = 962 ms; *SD* = 152 ms) (response option “False”) and inconsistent (*M* = 982 ms; *SD* = 168 ms) (response option “True”) conditions (*p* = 0.111), while the matching functional context showed faster RTs in consistent (*M* = 854 ms; *SD* = 131 ms) (response option “True”) compared to inconsistent (*M* = 962 ms; *SD* = 152 ms) (response option “False”) condition (*p* < 0.001). Finally, when we compared the two same response options “False” between consistent and inconsistent conditions, we found faster RTs in the matching functional context in the inconsistent condition (*M* = 926 ms; *SD* = 149 ms), compared to the mismatching functional context in the consistent condition (*M* = 962 ms; *SD* = 152 ms) (*p* = 0.006). While, when we compared the two same response options “True” between consistent and inconsistent conditions, faster RTs were found for the matching functional context in the consistent condition, compared to when it was mismatching in the inconsistent condition (*p* < 0.001).

Finally the interaction between Consistence and Hand Response was not significant *F*(1,22) = 0.157; *p* = 0.695; $\eta_{p}^{2}$ = 0.007; as well as between Functional Congruency and Hand Response *F*(1,22) = 0.251; *p* = 0.621; $\eta_{p}^{2}$ = 0.011 and between Consistence, Functional Congruency, and Hand Response *F*(1,22) = 0.02; *p* = 0.886; $\eta_{p}^{2}$ = 0.0009.

**FIGURE 3:**
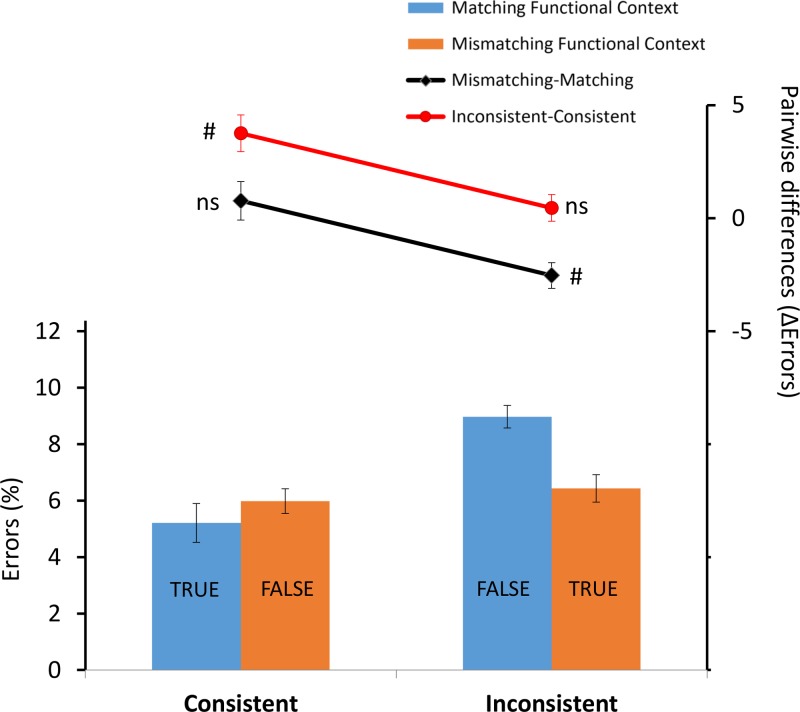
The bar plot shows the mean of percentage of error rates as a function of X (consistent vs. inconsistent) and Y (matching functional context vs. mismatching functional context) factors. Error bars indicate within-subjects SEM (Morey, 2008). The black and red line plots show, respectively, the functional context effect (i.e., the mismatching functional context – matching functional context pairwise difference) in consistent and inconsistent conditions and the consistency effect (i.e., the inconsistent–consistent pairwise difference) in matching functional context and mismatching functional context conditions. The corresponding error bars indicate the SEM of the pairwise differences (Franz and Loftus, 2012). (#) indicates consistency and functional context effects that were significantly (*p* < 0.05) different from 0; (ns) indicates non-significant effects. TRUE and FALSE represent the two response options.

The ANOVA on error rates revealed a significant main effect for Consistence *F*(1,22) = 16.19; *p* < 0.001; $\eta_{p}^{2}$ = 0.42 with more errors in the inconsistent condition (Mean = 7.7%, *SD* = 4.9%) compared to consistent (Mean = 5.6%, *SD* = 4.3%), and a significant interaction between Consistence and Functional Congruency *F*(1,22) = 11.93; *p* = 0.002; $\eta_{p}^{2}$ = 0.35 (**Figure 3**). *Post hoc* comparisons revealed more errors in the matching functional context for the inconsistent condition (*M* = 8.9%; *SD* = 5.1%) compared to all the other conditions (*p* < 0.001). No significant effect was found between matching (*M* = 5.2%; *SD* = 4.3%) and mismatching (*M* = 5.9%; *SD* = 4.5%) functional context in the consistent condition (*p* = 0.267) and between inconsistent (*M* = 6.4%; *SD* = 4.2%) and consistent conditions (*M* = 5.9%; *SD* = 4.5%) for the mismatching functional context (*p* = 0.510).

The remaining main effects and interactions were not significant: main effect of Functional Congruency *F*(1,22) = 2.67; *p* = 0.116; $\eta_{p}^{2}$ = 0.1; main effect of Hand Response *F*(1,22) = 0.05; *p* = 0.829; $\eta_{p}^{2}$ = 0.002; interaction between Consistence and Hand Response *F*(1,22) = 0.01; *p* = 0.908; $\eta_{p}^{2}$ = 0.006; interaction between Functional Congruency and Hand Response *F*(1,22) = 0.22; *p* = 0.645; $\eta_{p}^{2}$ = 0.009; interaction between Consistence, Functional Congruency, and Hand Response *F*(1,22) = 0.21; *p* = 0.649; $\eta_{p}^{2}$ = 0.009.

Mean and standard deviation of RTs and error rates of all effects are summarized in **Table 1**.

**Table 1:** Full reporting of analyses: mean (M) and standard deviation (SD) of reaction times (RTs) and the percentage (%) of error rates.

	Matching functional context		Mismatching functional context	
	Hand compatible	Hand incompatible	Hand compatible	Hand incompatible
**Consistent trials**				

Reaction times	*M* = 856 ms	*M* = 852 ms	*M* = 961 ms	*M* = 964 ms
	*SD* = 134 ms	*SD* = 134 ms	*SD* = 166 ms	*SD* = 149 ms
Error rates (%)	*M* = 5.2%	*M* = 5.1%	*M* = 5.9%	*M* = 5.9%
	*SD* = 4.5%	*SD* = 4.2%	*SD* = 4.4%	*SD* = 4.7%

**Inconsistent trials**				

Reaction times	*M* = 924 ms	*M* = 929 ms	*M* = 978 ms	*M* = 986 ms
	*SD* = 149 ms	*SD* = 153 ms	*SD* = 170 ms	*SD* = 175 ms
Error rates (%)	*M* = 9.2%	*M* = 8.7%	*M* = 6.3%	*M* = 6.5%
	*SD* = 5.2%	*SD* = 5.1%	*SD* = 4%	*SD* = 4.6%

## Discussion

We investigated the effect of consistent–inconsistent stimulus–response mappings, created by the IRAP, on the affordances activation given a matching–mismatching functional context. We expected that the matching functional context would induce faster RTs compared to the mismatching functional context. Consistent with our hypothesis and the previous experimental evidence on affordance given a functional context (Borghi et al., 2012; Wokke et al., 2016) our results showed that when participants related the label and the image stimuli, they were faster when the stimuli were functionally related (e.g., “Kitchen” – “Spatula”) than when they were not (e.g., “Kitchen” – “Hammer”).

In contrast to our hypothesis, we did not observe a difference in RTs when the orientation of the handle of the object matched participants’ hand response (spatial relation). Typically, affordance tasks consist of action decision or contextual/categorization tasks (Tucker and Ellis, 2001; Yoon et al., 2010; Goslin et al., 2012), in which, given an explicit instruction to focus on the objects, participants indicate the appropriate use for observed objects (action decision task), or their category (e.g., kitchen utensils) and context (e.g., kitchen environment). It has been argued that the action decision tasks are dependent on the *action knowledge*, whereas contextual decisions depend on access to *semantic knowledge*, and therefore they produce different effects on RTs (Yoon et al., 2010). For instance, faster RTs have been found during action decision tasks when the observed objects were co-located with the participant’s hand response, but this effect was widely reduced during contextual/categorization tasks.

In our task, participants “implicitly” learned to relate the stimuli and no explicit instruction to process the objects was given. Perhaps our task was functionally similar to contextual/categorization judgments, which require access to semantic rather than action knowledge.

An additional interpretation of our results is that the observation of tool-objects also induces a *“feature saliency effect”* (Kourtis and Vingerhoets, 2015) where participants’ attention is directed toward the functional part of the object (instead of the graspable part, *per se*) because the functional feature denotes the identity of the object. Indeed, this explanation would fit well with our results, if we consider that our task required contextual judgments (*“the spatula is usually found in the kitchen”*), instead of action judgments (*“I use the spatula in the kitchen”*). We argue that affordance effects modulated by spatial relations could be sensitive to the type of task we used. For instance, the alternation between consistent and inconsistent blocks could also have influenced the participants’ responses by reducing the impact of the spatial relations. This alternation may have worked as a top-down filter for affordance activation related to spatial relations. In this vein, the automaticity of affordance effects for spatial relations has been questioned and it is still being debated (for a review, see Borghi and Riggio, 2015). In fact, it has been shown that when participants performed a shape categorization task of the handles of the objects, the affordance effect was modulated by spatial relations (faster RTs when participants’ hand response was compatible with the orientation of the handles), but this effect disappeared when they performed a color categorization task (Tipper et al., 2006). Therefore, it seems that the automaticity of affordances activation for spatial relations depends on explicit instructions to process the objects.

Perhaps in our task, the use of words related to function verbs (e.g., “To cook”) instead of a functional context (e.g., “Kitchen”), would have had a different impact on the processing of spatial relations, in that it could help participants to focus on the *use* of the object rather than on its *identity*. The absence of a significant effect for the spatial relations does not mean that we did not find any affordance effect, where affordance in general is intended to mean the activation of potential actions induced by the observation of tools, which is shown (at a behavioral level) by a facilitation of RTs (faster RTs) in different contexts (Borghi and Riggio, 2015).

Moreover, in support of these variable results on spatial relations, Borghi et al. (2012) also proposed that spatial relations are sensitive to individual differences and less conventional, while functional relations are more conventional and socially established. In fact, we found that the functional (more conventional) relations did affect participants’ performance. Our results showed faster RTs for the matching functional context compared to when it was mismatching in both consistent and inconsistent conditions. More importantly, the functional context factor also interacted with the consistence factor of the stimulus–response mapping. One could expect that the conflict induced by the overlearned stimulus–response mapping could have a strong impact on affordances effects by slowing down the RTs independently from the functional context. But, when we compared the matching functional context between consistent and inconsistent conditions, we found faster RTs in consistent compared to inconsistent conditions, while we did not find a significant difference in RTs between consistent and inconsistent conditions when we compared the mismatching functional context.

This finding appears to be relevant to the argument that affordances can be modulated by *action semantics*, that is affordance processing is also determined by top-down influences related to the action context; (for a review, see van Elk et al., 2014). This means that when the functional relation between items mismatches, the action semantics are weakened and the consistent–inconsistent stimulus–response mappings have the same impact on participants’ behavior.

Interestingly, when participants were required to deny a conventional relation between stimuli that matched functionally (e.g., answer “False” for “Kitchen” – “Spatula”), the results showed faster RTs compared to when participants were required to deny a non-conventional relation between stimuli that did not match functionally (e.g., answer “False” for “Kitchen” – “Hammer”) (**Figure 2**). In other words, providing a conventional response for items that do not match functionally, induces slower RTs while providing an inconsistent response for a coherent functional context does not seem to slow down the participants’ performance, on the contrary induces faster RTs. This latter condition also affected the accuracy level compared to all the other conditions. Although participants had to reach a minimum of 80% accuracy on both consistent and inconsistent blocks of trials during a practice phase, and maintain this criterion during the critical test blocks, they provided less accurate responses exclusively in inconsistent blocks for the matching functional context. We interpret this result as indicating that there was a tendency for participants to respond automatically, in a conventional way and faster, when the functional relations between items were preserved although a non-conventional response was required.

Together these results on RTs and accuracy confirm our hypothesis regarding the dominance of the functional context on any stimulus–response mapping effect. In general, the results are compatible with previous findings that functional relations between stimuli modulate the responsiveness to affordances (Yoon et al., 2010; Borghi et al., 2012; Natraj et al., 2013; Wokke et al., 2016). But in addition, the current data support the conclusion that this modulatory effect is maintained even if a manipulation of stimulus–response mappings interferes with the required actions.

One could also argue that the simple semantic relations between the stimuli would have led to the described effects, which could be seen for any pair of related items, whether or not they were even graspable objects. However, it has been shown, for instance that the simple observation of words that are semantically related but also representing features related to the functional use of an object (e.g., *thirst-cup; nail-hammer*) led to a congruency effect (faster RTs), which disappeared when the words denoted a less functional use (e.g., *sink-cup*; *tool belt-hammer*) but were still semantically related (van Dam et al., 2010). Therefore, semantically related items induced faster RTs specifically when they denoted only a functional use. At the same time, the sight of graspable objects (“pan”) in combination with function verbs (“to cook”) induced the same congruency effect, compared to observation verbs (“to look at”) and only when they were presented in the reachable space (Costantini et al., 2011). This evidence supports the view that the action possibilities are only elicited by the functional context/use.

Although, this evidence could be seen as consistent with our findings, by suggesting that the processing of a matching functional context may activate a simulation of potential interactions/actions with the graspable objects despite the conflict of required non-conventional responses (e.g., to deny a congruent functional use for the observed stimulus), we cannot fully exclude the influence of semantic relations in our results. Therefore, a limitation of our work is that our task does not allow to distinguish completely the role of semantic information about the objects, from the functional use of objects since we did not find any significant hand-response compatibility effect. It could be possible that the semantic relations of the stimuli had a strong impact on the participants’ performance. We believe that the requirement in our task to attend to both the functional context and the consistency versus inconsistency of the stimulus–response mappings may have worked as a top-down filter for affordance activation related to spatial relations. But, this latter aspect requires additional investigations.

Overall, we show for the first time a modulation of RTs and accuracy in the context of tool-objects processing and language when non-conventional responses are required. We therefore, propose a “*fluency mechanism*” for affordances activation that drives motor responses when the functional relations are preserved within conflicting situations.

Certainly, future research need to be addressed to prove the role of motor system for affordances processing within this framework. A valid option could be to focus on electrophysiological correlates to test the time course of affordances processing for consistent–inconsistent stimulus–response mappings of functional relations, by considering both motor (e.g., readiness potentials) and action-semantic (e.g., N400) event related potentials. This may reveal if the functional context is operating as an early or late filter for affordances activation related to spatial relations, and provide evidence on how the motor system is involved for the processing of stimuli that are functionally related within conflicting circumstances.

## Author Contributions

RV and MF conceived and designed the study. MF collected the data. RV analyzed the data. DB-H supervised the research and gave important contribution for drafting the final version of the manuscript. All authors contributed to write the manuscript and approved the final version.

## Conflict of Interest Statement

The authors declare that the research was conducted in the absence of any commercial or financial relationships that could be construed as a potential conflict of interest.
